# Resilience and Associated Factors in Mothers of Children with Congenital Heart Disease: A Cross-Sectional Study

**DOI:** 10.30476/ijcbnm.2021.89691.1630

**Published:** 2021-10

**Authors:** Masomeh Sanayeh, Manijeh Nourian, Saleheh Tajalli, Fatemeh Khoshnavay Fomani, Amir Heidari, Malihe Nasiri

**Affiliations:** 1 Department of Pediatric Nursing, School of Nursing and Midwifery, Shahid Beheshti University of Medical Sciences, Tehran, Iran; 2 Nursing Care Research Center (NCRC), School of Nursing and Midwifery, Iran University of Medical Sciences, Tehran, Iran; 3 Department of Pediatric Nursing, School of Nursing and Midwifery, Tehran University of Medical Sciences, Tehran, Iran; 4 Department of Cardiovascular, School of Medicine, Shahid Beheshti University of Medical Sciences, Tehran, Iran; 5 Department of Biostatistics, School of Nursing and Midwifery, Shahid Beheshti University of Medical Sciences, Tehran, Iran

**Keywords:** Children, Congenital Heart Disease, Mothers, Optimism, Resilience, Social support

## Abstract

**Background:**

Congenital heart disease (CHD) in children affects the parents’ mental and physical health and their caring and supportive functions. Resilience is an important factor in health. This study aimed to determine resilience and its associated factors in mothers of children with congenital heart disease.

**Methods:**

This descriptive cross-sectional study enrolled 136 mothers of children with CHD. Samples were selected in two hospitals of Shahid Modarres and Children’s Medical Center in Tehran, Iran, via convenience sampling from May to early October 2019. Data were collected using demographics questionnaire, Multidimensional Scale of Perceived Social Support (MSPSS), Life Orientation Test (LOT), and Wagnild and Young Resilience Scale (RS). Data were analyzed in statistical software SPSS-16 using independent t-test, ANOVA, Pearson and Spearman correlation, and multiple linear regression. The significance level was set at P<0.05.

**Results:**

Mothers’ total mean score of resilience was 94.08±12.81, while 14.7%, 66.2%, and 19.1% had low, moderate, and high resilience, respectively. Regression analysis showed that two variables of social support (B=2.20, P<0.001) and optimism (B=0.76, P=0.003) were associated with resilience in mothers. The duration of the child’s disease was positively correlated with the mothers’ resilience but did not predict it (B=-0.72, P=0.22).

**Conclusion:**

The level of resilience in most mothers was moderate. Social support in the dimension of significant others and optimism were associated with mothers’ resilience. Therefore, holistic care and providing educational training programs that enhance these variables could be effective in promoting resilience in mothers of children with CHD.

## INTRODUCTION

Congenital heart disease (CHD) is the most common birth defect and its incidence is constantly increasing. ^1^ The CHD incidence is high in developing countries in Asia and has been increasing progressively from 1970 to 2017, reaching a maximum of 9.4/1000. ^1^ This chronic disease is characterized by structural and functional defects in the heart and large vessels. ^1^ CHD exposes children to physical and neurodevelopmental problems, increases the parents’ stress, and leads to a decrease in their quality of life. ^2^

Parents of children with CHD are more stressed than those of children with other chronic diseases. ^3^ In most societies, child care is the responsibility of mothers ^4^ and their stress levels have been reported high. ^5^ This chronic stress is present from diagnosis to hospitalization and during the child’s surgery, or even months and years later. ^3 , 5^ Evidence indicates that only 56.7% of mothers of children with a chronic illness are in good physical and emotional health. ^6^ This figure has also been reported lower for parents of children with CHD. ^7^ These mothers are at risk of anxiety, depression, restlessness, uncertainty, guilt, and failure to communicate with the child, which harms the mental and physical health and caring and supportive functioning of mothers and families, ^2 , 8^ as well as the process of child’s treatment and improvement. ^4^

These mothers need support to increase their level of positive adaptation. Resilience is defined as the capacity to deal with and recover from adversity ^9^ and is one of the most important components affecting health that results in positive adaptation to difficult living conditions, prevents negative consequences of facing predicaments and life stresses, improves the quality of life, and promotes satisfaction and mental and social health. ^10 , 11^

Resilience is a phenomenon that depends on the context of life and can be affected by personal, family, and social factors in mothers of children with CHD. Applied research has introduced optimism as an individual factor that promotes positive feelings and outcomes. ^12^ The authors of the present study found no study on the predictive value of optimism in the resilience of mothers of children with CHD. However, the results of some studies approved a positive correlation between optimism and resilience, while some others reported a negative relationship between optimism and resilience. ^13 , 14^ Furthermore, parents of children with CHD have uncertainties about the child’s illness and life in future. ^15^ This factor can increase stress and decrease hope in the future and can make the relationship between optimism and resilience ambiguous. 

Social support plays an important role in enhancing well-being and health and has a positive effect on resilience; ^16 , 17^ however, some experts believe that receiving social support is not a predictive variable of resilience. ^18^ A study also has shown that resilience is influenced by the structure of a person’s “self” rather than external factors such as social support or economic status. ^19^ On the other hand, although social support can be effective in reducing negative psychological and family consequences in parents of children with CHD, ^3^ its effect on the resilience of the group of mothers living in a context of care and parental stress is not clear, especially when various factors such as the level of distress and despair, the high costs of illness, as well as the long duration of care provision in these mothers, may reduce their perceived social support. 

There are few studies on the resilience of mothers of children with CHD and its related factors in Iran and studies in this area have been mostly in Western societies. This study aimed to determine resilience and its associated factors such as individual characteristics, social support, and optimism of mothers of children with CHD, as well as the personal and clinical characteristics of the children.

## MATERIALS AND METHODS

This cross-sectional study was conducted in Pediatric, Surgical, and Pediatric Cardiac wards in Shahid Modarres and Children’s Medical Center hospitals affiliated to Shahid Beheshti and Tehran University of Medical Sciences, Tehran, Iran from May to October 2019. The target population consisted of mothers of children with CHD. Participants were selected via convenience sampling. The inclusion criteria were having a child with CHD less than 6 years old who is most dependent on his/her mother for care; the child being definitely diagnosed with CHD by a cardiologist; being the child’s biological mother; mother’s being aware of the child’s illness; mother’s being the primary caregiver; being literate, mothers not having a history of chronic mental and physical illnesses based on their medical record; having physical, cognitive and speech ability to complete the questionnaires; and not having experienced other stressful events such as divorce, child death, etc. in the past 6 months for the mother. Participants who had not fully completed questionnaires were excluded. The sample size was determined by considering the formula below and a previous study. ^10^

$\frac{Z_{1-\alpha /2}^{2}\times \sigma^{2}}{d^{2}}$

σ=11 z=1.96 α=0.05 d=2

The minimum sample size of 116 was obtained; also, considering 15% of the sample loss, 134 samples were considered. A total of 142 mothers were asked to participate in the study, of whom 6 were excluded due to failing to fill out the questionnaire completely or returning them. Finally, the data of 136 mothers were analyzed, which was two mothers more than the sample size calculated by the above formula.

 Data were collected by the first author and a nurse who had a similar level of education and was trained in the objectives and methodology. The mothers completed the questionnaires up to 24 hours before the surgery or any cardiac catheterization in a quiet room. Questionnaires took 20-25 minutes to complete. The researcher without any interference in completing the questionnaires was present when the mothers completed the questionnaires and answered their questions. The personal characteristics questionnaire was completed by the researcher using medical records and interviews with mothers. The sampling period lasted from May to early October 2019. 

Data were collected using four questionnaires:

1. Demographic characteristics questionnaire on the mother’s age, child’s age, duration of child’s disease, mother’s occupation, number of children, child’s sex, type of disease, child’s birth order, mother’s education level, frequency of hospitalization, type of child’s insurance, and the history of the disease in other family members. 

2. Multidimensional Scale of Perceived Social Support (MSPSS) which has three 3 subscales (family, friends, significant others) and twelve statements scored on a 7-point Likert scale (from one to seven). Its score ranges from 12 to 84, and higher scores indicate greater overall perceived social support. ^20^ MSPSS has been translated into different languages and has been proved valid and reliable. ^21 , 22^ Its Persian version has been used in various studies. ^23^ A psychometric study reported the three-factor structure of the Persian version of the tool and its Cronbach’s alpha was 0.84 for the whole tool and 0.85-0.93 for its subscales. ^24^ In the present study, the reliability of MSPSS using Cronbach’s alpha coefficient for the whole tool was 0.88 and its reliability was between 0.78-0.84 in all sub-scales. 

3. Life Orientation Test (LOT) which was designed by Scheier et al. in 1994 with ten statements scored on a 5-point Likert scale (0 to 4). Four items in this tool (items 2, 5, 6, and 8) are not scored. Its total score is between zero and 24, with scores above 12 indicating optimistic orientation and those below 12 indicating pessimistic orientation. ^25^ This tool has been used in various studies in Iranian communities. ^26 , 27^ The psychometric properties of its Persian version were reviewed by Kajbaf et al. (2006) and its reliability (0.74) and repeatability (0.87) were acceptable. ^27^ In the present study, reliability was calculated as Cronbach’s alpha of 0.86. 

4. Wagnild and Young Resilience Scale (RS) which examines the resilience construct as an individual characteristic on a five-point Likert scale (one to five). It has two factors and 25 items (Personal Competence with 17 statement and Acceptance of Self and Life with 8 statement), and the total score was between 25 and 125. Higher scores indicate more resilience. Resilience is accordingly scored as very low (<60), low (60-85), moderate (86-105), and high (106-125). ^28^ The psychometric study of the Persian version of this tool among Iranian adolescents reported reliability of 0.77 and an intra-class correlation coefficient of 0.86 in 2016. ^10^ In the present study, the reliability of the instrument was 0.88 in all factors, 0.83 for Personal Competence, and 0.77 for Acceptance of Self and Life.

The qualitative content validity of all the three questionnaires (MSPSS, LOT and RS) was approved by 11 social sciences, nursing, and psychology academic members in Shahid Beheshti University of Medical Sciences.

The present study was approved by the Ethics Committee (IR.SBMU.PHARMACY.REC.1397.101) in Shahid Beheshti University of Medical Sciences. All participants were informed of the study objectives; they signed a written informed consent form and were assured of the confidentiality of their personal information and the voluntary nature of participation.

Data were analyzed in SPSS-16 using descriptive statistics (Mean, Frequency and Percentage) and the method of multiple linear regression (for assessing the variables associated with mothers’ resilience), Pearson’s and Spearman’s correlation coefficient (for determining associations between variables), t-test, and one-way ANOVA. Results of the Shapiro-Wilk test showed that only two variables (Duration of child’s disease and Child’s age) did not have a normal distribution (P<0.05). The significance level was set at α=0.05.

## RESULTS

Out of 142 mothers who consented and entered the study, 6 were excluded due to missing data (95.7%). The mean age of the mothers and children was 29.54±3.98 and 3.56±1.30 years, respectively. The personal characteristics of the mothers and children are presented in Table 1. The mean of the mother’s resilience was 94.08±12.81 (Table 2). Among the mothers, 14.7% had low resilience, 66.2% had moderate resilience, and 19.1% had high resilience. Independent t-test and one-way ANOVA tests were used to compare the mean score of resilience in terms of personal and clinical variables (Table 1). 

**Table 1:** Resilience score among different characteristics of mothers/children (n=136)

Variables	Number (%)	Mothers Total resilience score Mean±SD	P value
Mother’s education level			0.76*
High school^a^	14 (10.29)	93.92±7.96	
Diploma^b^	41 (30.15)	95.27±14.71	
College	81 (59.56)	93.68±12.88	
Mother’s Occupation			0.87*
Housewife	84 (61.76)	94.45±13.08	
Employee	27 (19.85)	94.00±11.64	
Others	25 (18.38)	92.92±13.51	
Number of children			0.96**
1-2	73 (53.67)	93.69±13.07	
3 and more	63 (46.33)	65.94±11.72	
History of the disease in other family members			0.08*
No record	76 (55.89)	98.20±11.1	
1 record	44 (32.35)	99.10±12.6	
2 records	16 (11.76)	99.90±11.2	
Child’s birth order			0.08*
The first child	103 (75.74)	93.28±12.76	
Second and Third	21 (15.44)	99.62±12.65	
The last child	12 (8.82)	91.25±11.76	
Child’s sex			0.13**
Female	72 (52.94)	95.83±12.49	
Male	64 (47.06)	92.53±12.98	
Type of child’s insurance			0.81*
Health insurance	48 (35.29)	92.20±9.92	
Social insurance^c^	72 (52.94)	91.17±11.22	
others	16 (11.76)	92.10±10.50	
Type of Disease			0.28*
Atrial septal defect	21 (15.45)	96.48±12.62	
Ventricular septal defect	23 (16.92)	92.70±15.32	
Aortic Coarctation	24 (17.64)	98.17±13.06	
Tetralogy of Fallot	28 (20.59)	91.36±18.64	
Arterial stenosis	16 (11.76)	94.75±12.70	
Aortic stenosis	24 (17.64)	90.56±10.09	
Frequency of hospitalization			0.82**
1-2	87 (63.97)	93.90±12.83	
3-4	49 (36.03)	94.49±12.71	

a High school: From the first to the third year of high school;

b Diploma: Someone who has won a diploma;

c Social insurance: is one of the types of health insurance;

* one-way ANOVA,

** t-test, P<0.05

**Table 2:** Resilience mean and its dimensions in mothers of children with Congenital Heart Disease

Variable Mother’s resilience	Mean±SD	Minimum	Maximum
Acceptance of Self and Life	30.64±3.95	22.00	40.00
Personal Competence	63.43±9.20	43.00	85.00
Total	94.08±12.81	44.00	125.00

The variables of mother’s optimism (r=0.27, P=0.006), mother’s social support in three dimensions (r=0.29 P=0.001, r=0.36 P<0.001, r=0.61 P<0.001) and duration of the disease (r=0.20, P=0.01), which were significantly correlated with the mothers’ resilience (Table 3), were selected and entered into the regression model. Mother’s total social support score (r=0.50, P<0.001) was not entered into the regression because it could create a co-linearity problem. The results of regression analysis showed that two variables of social support in the dimension of significant others (P<0.001, B=2.20) and optimism (P=0.003, B=0.76) were associated with resilience in mothers of children with CHD. Each unit of increase in the social support by significant others increased the mothers’ resilience score by 2.2 units on average and each unit increase in optimism led to 0.76 unit of increase in mothers’ resilience score on average (Table 4).

**Table 3:** Factors related to mothers/children characteristics and the resilience scores of mothers

Variables	Mean±SD	Resilience score(r)	P value*
Mother’s age(years)	29.54±3.98	0.13	0.11**
Child’s age(years)	3.56±1.30	-0.01	0.82*
Duration of child’s disease (years)	2.69±1.46	0.20	0.01*
Mother’s optimism	18.49±2.98	0.27	0.006**
Mother’s Social Support (total)	51.98±7.55	0.50	<0.001**
Mother’s Social Support (family)	17.89±3.30	0.29	0.001**
Mother’s Social Support (friends)	17.90±3.46	0.36	<0.001**
Mother’s Social Support (significant others)	17.60±3.64	0.61	<0.001**

* Spearman correlation;

** Pearson correlation

**Table 4:** The multiple linear regression analysis with the mother’s resilience as dependent variable

Model	Non-standardized		Standardized	t	P value
	B	Std Error	Beta		
Constant	70.72	7.45		9.48	<0.001
Duration of child’s disease	-0.72	0.61	-0.08	-1.17	0.24
Mother’s Social Support (family)	-0.23	0.34	-0.06	-0.67	0.50
Mother’s Social Support (friends)	0.18	0.32	0.05	0.58	0.56
Mother’s Social Support (significant others)	2.20	0.34	0.62	6.39	<0.001
Mother’s optimism	0.76	0.28	0.17	2.71	0.003

## DISCUSSION

The results showed that the level of resilience in most mothers was moderate. Two variables, social support in the dimension of significant others and mothers’ optimism, were associated with mothers’ resilience. More than half of the mothers had moderate resilience and very few of them were found to be low in resilience. The authors did not find a study that examined resilience in this group of mothers. A study showed nearly half of the family caregivers of patients with mental disorders had moderate resilience (good resilience). ^29^ Another study on parents of children with cancer in China reported an average level of resilience and tenacity. ^30^ Unlike the present study, another study on the resilience of parents of children who had developed chronic diseases such as thalassemia ^31^ and cerebral palsy ^32^ reported high resilience. This difference in results may be due to differences in the type of children’s chronic diseases and different methodologies. 

Parents’ exposure to the problems associated with the care and treatment of a child with a chronic illness can improve parental resilience over time. The results of a study showed that parents of these children face many difficulties such as going through screening and diagnosis stages, potentially life-threatening interventions, multiple surgeries, developmental delays, and death or near-death experiences. ^33^ Besides, the experiences of parents of children who were a candidate for CHD surgery showed that parents experienced feelings of helplessness, uncertainty, fear, and sadness from diagnosis throughout their child’s life. ^4 , 8^ Such situations and the difficult living conditions can promote resilience and emergence of strength in some mothers. ^34^

According to the results, none of the personal characteristics of children and mothers was associated with the effect of resilience in the present study. Consistent with these results, a descriptive study on 220 family caregivers of patients with a mental disorder in Iran showed that there was no statistically significant relationship between the caregivers’ resilience and their characteristics. ^29^ There was a significant positive correlation between the duration of a child’s illness and mother’s resilience in this study, indicating that mother’s resilience increased with the duration of the child’s illness, but the results of regression analysis showed that the duration of the disease was not associated with mother’s resilience. A study also found that the duration of a child’s cancer did not play a role in predicting parental resilience. Inconsistent with the results of the present study, the above study reported a child’s male sex associated with less parental resilience. ^35^ This inconsistency can be due to different types of diseases in the above-mentioned as well as the present study. Furthermore, the above study examined resilience in parents (father and mother), and it should be noted that in Asian countries parents spend most of their time with their sons, so son’s cancer can be associated with lower resilience in parents, especially fathers.

The results of the present study on the presence of a strong, positive and significant correlation between perceived overall social support and mothers’ resilience are supported by various studies. ^23 , 36^ A qualitative study on family resilience of patients admitted to intensive care unit reported that the family support system is one of the important facilitators of family resilience, and the way each family member is supported is also important. ^17^

Results of this study showed that social support by significant others (Important and influential people in mother’s life) had the highest association with the resilience of mothers of children with CHD such that each unit increase in the social support by significant others increased the mothers’ resilience score by 2.2 units on average. An extensive network of relationships for support can increase confidence and reduce anxiety in mothers, leading to an increase in resilience. Social support is a strong coping force for successful and easy dealing with chronic illnesses and stressful conditions for parents. ^37^ Social support can decrease the mother’s psychological stress, and help perceive stressful events as less threatening by protecting the mother against stressors of exposure to the child’s disease and promoting the parents’ positive adaptation by reducing stress and meeting their needs. ^37 , 38^ This finding is similar to the results of a study in Ethiopia that showed receiving support from friends is a positive predictor of resilience in parents of children with cancer. ^39^

Having a child with chronic illness can affect the quality of life of the family, especially the mother. By emphasizing the role of social support to enhance the resilience and quality of life of mothers, it is possible to improve the quality of life of the sick child and accelerate the process of recovery and treatment. ^3^ The results of research on parents of children with CHD showed that perceived social support is directly correlated with the parents’ quality of life. ^40^ Mothers who receive sufficient social support find their lives meaningful and do not feel alone in the face of problems. They reduce the effect of stresses on themselves by feeling attached to the people that support them; thus, they experience lower parental stress, and thereby improve the level of health. ^41^

In this study, the mothers’ optimism had an association with the mothers’ resilience as a unit increase in optimism led to a 0.76 unit increase in mothers’ resilience score on average. Several studies have approved the predictive role of optimism in resilience and its positive effect as an adaptive strategy on caregiving stress in these families. ^42 , 43^ One of the foundations of positive psychology and optimism is nurturing purpose and meaning in life, ^44^ and mothers who are optimistic improve their resilience by increasing the meaning of life and setting targets for themselves. Optimism can prepare one’s mind to choose appropriate and creative ways to move forward and solve problems, and enhance the ability to adapt positively to the hardships of life. ^42^ Optimism, a positive look at life, and hope for better conditions help the mothers manage their life problems and positively adapt to problems while maintaining positive physical and mental health. 

The present study recruited the mothers of children with CHD in two hospitals of Shahid Modarres and Children’s Medical Center with convenience sampling and the results should be cautiously generalized. Another limitation of this study was the self-report nature of data collection tools; a large number of questionnaires could affect the accuracy of the participants’ answers. The other limitation was that the fathers were not included in the study. On the other hand, longitudinal data are more appropriate for predictive studies. Regardless of the limitations, this study is the first one in Iran to examine the resilience of mothers of children with CHD, and this issue can be its strength and the results will provide the basis for further studies. Improving the resilience of mothers of children with CHD is important in nursing practice to reduce the mothers’ stress and increase their positive adaptation and quality of life. The findings of the present study can be effective to plan and implement health-promoting interventions to improve the quality of life of children with CHD and their families.

## CONCLUSION

The level of resilience in most mothers was moderate. For enhancing social networks, more attention is needed to assess the mothers’ current social support systems and provide holistic care based on their individual needs, resources, and cultural considerations. Teaching mothers new skills for improving their communication can be useful. Optimism interventions must be tailored based on educational training programs. Providing optimism training in the form of group counseling, focusing on family competencies, and using educational booklets can also be helpful. It is recommended that further longitudinal studies consider the moderating role of mothers’ and children’s variables in the predictive effect of social support and optimism.
